# The current understanding of KRAS protein structure and dynamics

**DOI:** 10.1016/j.csbj.2019.12.004

**Published:** 2019-12-26

**Authors:** Tatu Pantsar

**Affiliations:** a Department of Pharmaceutical and Medicinal Chemistry, Institute of Pharmaceutical Sciences, Eberhard Karls University Tübingen, Auf der Morgenstelle 8, 72076 Tübingen, Germany; b School of Pharmacy, University of Eastern Finland, Yliopistonranta 1, 70210 Kuopio, Finland

**Keywords:** KRAS, Ras Proteins, Proto-Oncogene Proteins p21(ras), Cancer, Molecular Dynamics Simulation, Protein conformation, Drug discovery

## Abstract

One of the most common drivers in human cancer is the mutant KRAS protein. Not so long ago KRAS was considered as an undruggable oncoprotein. After a long struggle, however, we finally see some light at the end of the tunnel as promising KRAS targeted therapies are in or approaching clinical trials. In recent years, together with the promising progress in RAS drug discovery, our understanding of KRAS has increased tremendously. This progress has been accompanied with a resurgence of publicly available KRAS structures, which were limited to nine structures less than ten years ago. Furthermore, the ever-increasing computational capacity has made biologically relevant timescales accessible, enabling molecular dynamics (MD) simulations to study the dynamics of KRAS protein in more detail at the atomistic level. In this minireview, my aim is to provide the reader an overview of the publicly available KRAS structural data, insights to conformational dynamics revealed by experiments and what we have learned from MD simulations. Also, I will discuss limitations of the current data and provide suggestions for future research related to KRAS, which would fill out the existing gaps in our knowledge and provide guidance in deciphering this enigmatic oncoprotein.

## 1 Introduction

GTPase KRas (KRAS) is a signal transducer protein, which plays an important role in various cellular signalling events such as in regulation of cell proliferation. It is a critical hub in the cell circuitry, as upon an upstream stimulus it transduces activating signals to several cellular signalling pathways, including the mitogen-activated protein kinase (MAPK) pathway [1]. KRAS cycles between inactive guanosine diphosphate (GDP)-bound and active guanosine triphosphate (GTP)-bound states [2]. Only in the GTP-bound state, KRAS is able to bind and activate its effector proteins, such as RAF-kinases, PI3K and RalGDS. KRAS itself becomes activated when a guanosine exchange factor (GEF) protein displaces GDP from the nucleotide binding site, resulting eventually in GTP binding, as there is a higher intracellular concentration of GTP than GDP [3]. Inactivation of the active KRAS occurs upon GTP hydrolysis to GDP. On its own KRAS has low intrinsic GTPase activity, which is greatly enhanced by GTPase activating proteins (GAP) that catalyse the hydrolysis reaction [3], [4].

Hyperactivation of RAS signalling, which may occur via a direct mutation of RAS or indirectly via other proteins in RAS pathways, plays a significant role in cancer and in particular rare diseases such as RASopathies [5]. There are three closely related RAS isoforms: HRAS, KRAS and NRAS. From all of the RAS isoforms, KRAS is the most oncogenic with its 85% share of all mutated RAS proteins observed in cancer [5], [6]. KRAS missense mutations are particularly frequent in the pancreatic, colorectal and lung cancers (COSMIC v.90) [7]. In cancer, three mutation hotspots: G12, G13 and Q61 are observed in RAS genes. In this regard, KRAS differs from the NRAS and HRAS, as it is the only RAS isoform where the position 12 mutations are predominant [6].

**Fig. 1:**
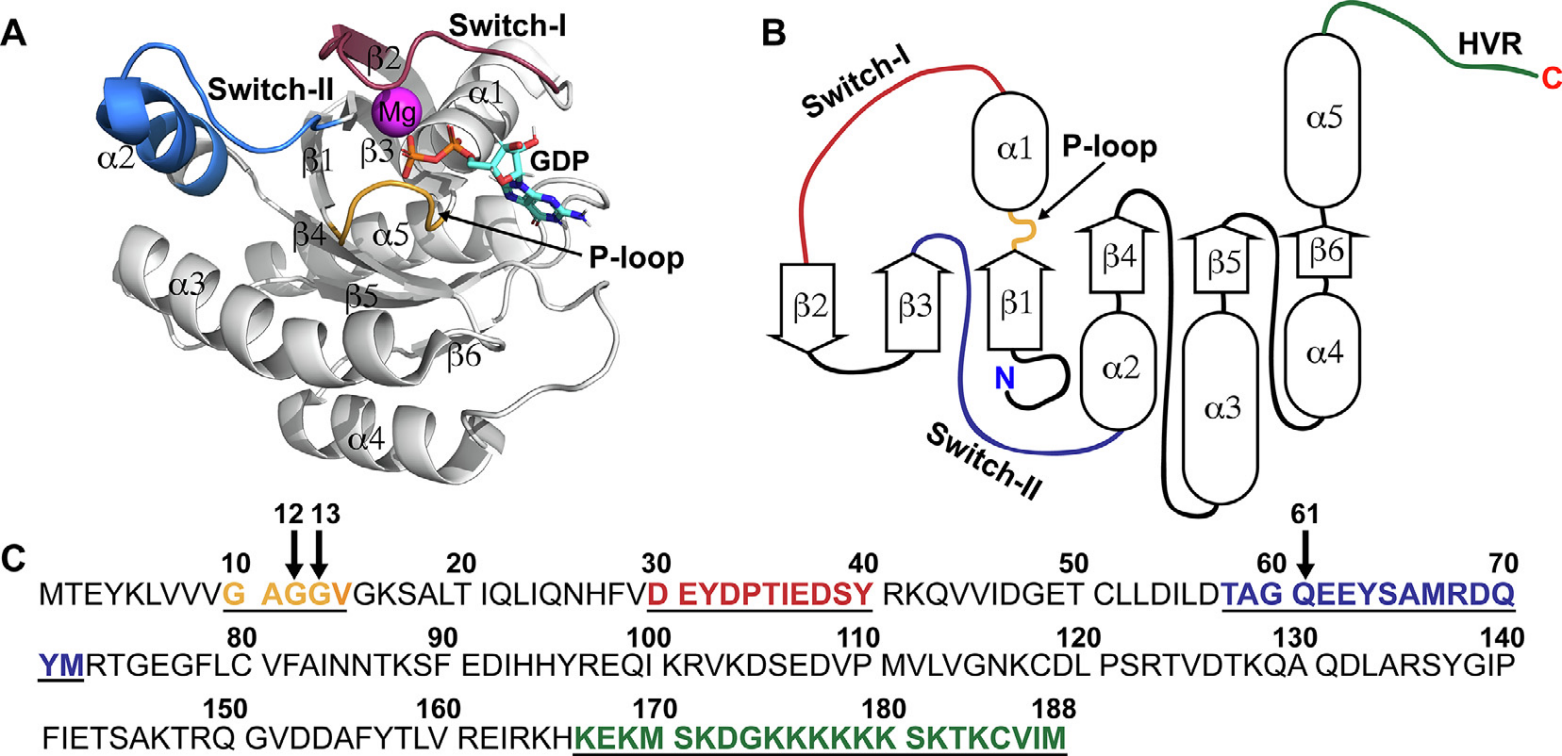
Structure and sequence of KRAS4B. (A) Crystal structure of wild-type (WT) KRAS with GDP-bound (PDB ID: 4obe) [14]. The C-terminal HVR is not present in the structure (residues 1–169 were used in the protein construct). (B) 2D depiction of the secondary structure of KRAS. (C) Sequence of KRAS4B, also known as isoform 2B (Uniprot: P01116-2). The most common mutation hotspots are depicted with arrows. Selected structural regions in all A–C highlighted with the following colour scheme: P-loop (residues 10–14), orange; switch-I (residues 30–40), red; switch-II (residues 58–72), blue; HVR (residues 167–188), green. (For interpretation of the references to colour in this figure legend, the reader is referred to the web version of this article.)

The G domain of KRAS, comprised of residues 1–166 (Fig. 1A), forms the basis of biological functionality of the GTPase proteins [8]. This domain encompasses six beta-strands, forming the protein core, surrounded by five alpha-helices (Fig. 1B). In addition to the G domain, KRAS has a flexible C-terminal structural element, named the hypervariable region (HVR), which plays a crucial role in anchoring RAS to the membrane [9], [10], [11]. Other important functional elements of KRAS are the switch-regions, so-called switch-I and switch-II. These switches form the binding interface for effector proteins, as well as for RAS regulators (GAPs and GEFs). To point out, several residue definitions are used for the switch regions in the literature, which are rather arbitrary, due to high intrinsic flexibility of these regions. For instance, in switch-II definitions, the beginning falls between residues 58–60 and ends among residues 67–76, excluding or including partially or fully the helix α2. Here, for the illustrative purposes only, a definition of residues 30–40 for switch-I, residues 58–72 for switch-II and residues 10–14 for P-loop is used (although P-loop, also known as Walker A motif [12], extends to the S17 [13]). The mutation hotspots in cancer are located in P-loop or in switch-II (Fig. 1C).

The gene *KRAS* may undergo alternative splicing and thus result in two isoforms: KRAS4A and KRAS4B (also known as isoform 2A and 2B, respectively). These isoforms differ mainly in their HVR residues 167–189, but also residues 151, 153, 165 and 166 are dissimilar.

Active KRAS signalling occurs at the membrane. In order to become associated to membrane, KRAS’ membrane anchoring HVR needs to undergo a few post-translational modifications [15]. First, the C-terminal CAAX sequence (CVIM in KRAS4B) is farnesylated at C185, which is followed by proteolytic cleavage of the three terminal residues. Finally, the terminal carboxyl group of C185 is methylated. A polybasic region of the HVR, composed of multiple lysine residues, is also important for the membrane association [9]. As KRAS4A does not contain this polybasic region, it is further palmitoylated at an additional cysteine residue C180 [15].

Also, other post-translational modifications of KRAS have been described. For instance, phosphorylation of S181 was demonstrated, which influences to KRAS interaction with Calmodulin (CaM) and also to tumour growth [16], [17]. Monoubiquitination of K147, which is located in the nucleotide binding site, was shown to increase KRAS’ activity [18]. Furthermore, KRAS acetylation was observed at lysine residues K101, K104, K128 and K147 [19], [20]. Recently, excision of the initiator methionine (M1) accompanied with acetylation of the N-terminal threonine (T2) was disclosed [21]. The acetylation of T2 appears important for switch stability upon the excision of M1 residue, which by itself makes the N-terminus unstable.

Due to its crucial role in cancer biology, KRAS is sometimes referred as the Holy Grail of drug discovery [22]. Formerly, it was considered as an undruggable protein, but now is rather cogitated as a challenging target, which is difficult to drug [23]. Currently, Amgen’s KRAS G12C inhibitor AMG 510 is in clinical trials [24], [25]. Recent substantial progress in KRAS drug discovery, however, is limited to G12C-specific inhibitors, excluding other oncogenic KRAS mutants that form the majority in other tissues than in the lung [26], [27]. In fact, we still do not fully understand the underlying reasons of specific mutation frequencies [28]. Discrepancy in KRAS mutations exist, *e.g.* in their GTP hydrolysis rates, and even mutations at the same position display tissue-specific abilities to drive tumorigenesis *in vivo* [4], [29], [30]. These complex issues and lack of understanding of the underlying principles, still require major efforts to be resolved in future.

In order to tackle these issues, thorough understanding of KRAS structure and dynamics comes into play. This could provide an extra leverage to the drug discovery efforts against KRAS. The aim of this minireview is to provide an overview of the current understanding of KRAS structure and conformational dynamics. Furthermore, limitations of the structural data regarding to this matter are highlighted and lessons learned from molecular dynamics (MD) simulations are revealed. Finally, future perspectives regarding to structural and dynamical aspects of KRAS are discussed.

## 2 Publicly available KRAS structural data

**Fig. 2:**
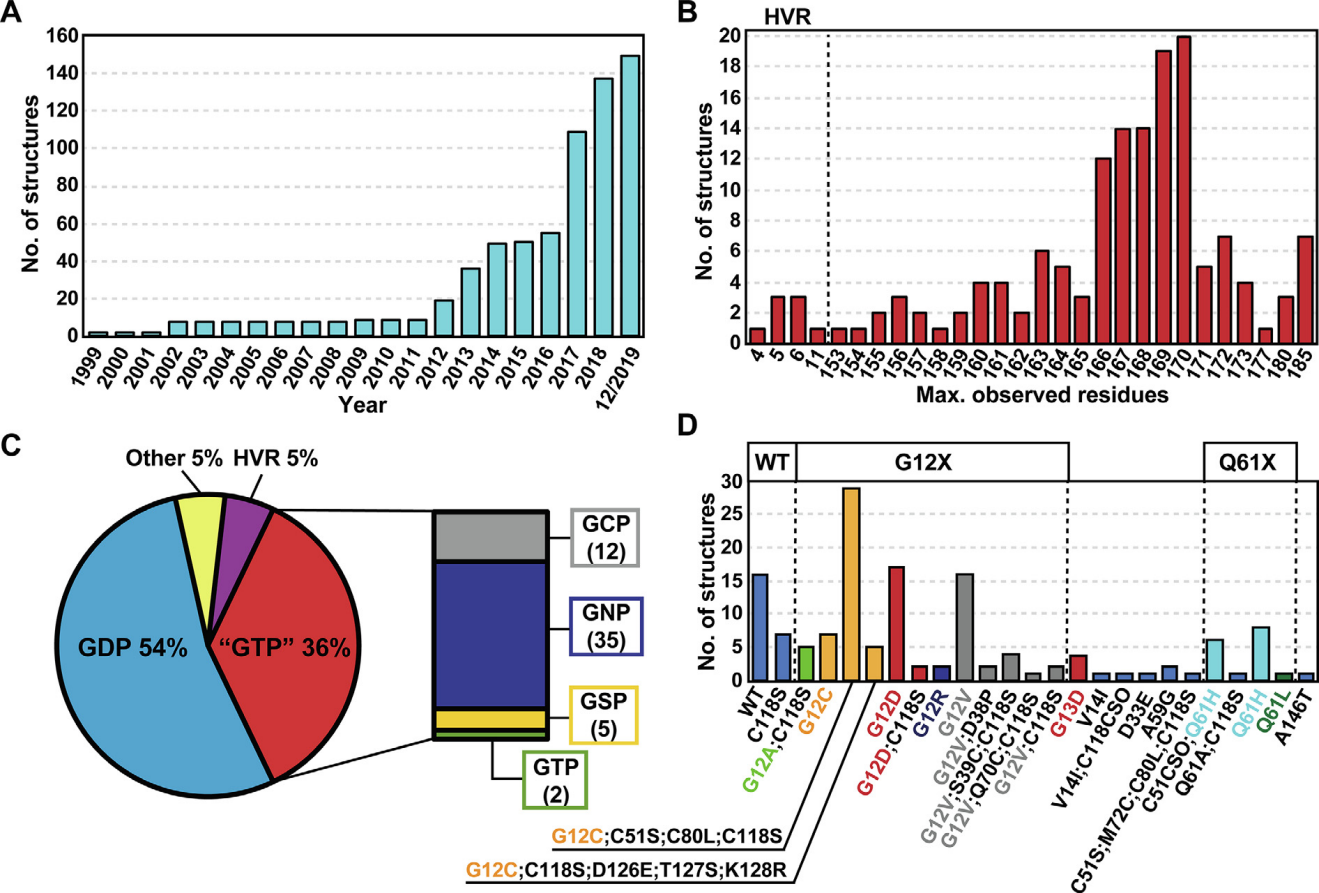
Publicly available KRAS structural data in the Protein Data Bank. (A) Timeline of the evolution of publicly available KRAS structures. First KRAS structures were deposited in 1999 and a sharp increase in the number of structures has occurred during the recent years. In 2017, the number of KRAS structures doubled and since dozens of structures have been deposited yearly. (B) The maximum number of observed residues in the available KRAS structures. Eight structures describe only the HVR-region of KRAS, whereas in the majority of structures only the G domain is present and the HVR region is disordered or was not present in the protein construct. Note that six of the full-length (185 residues) structures are NMR-data driven models and the only crystal structure where all residues are observed is from the KRAS–PDEδ complex (PDB ID: 5tar), where the HVR is stabilized by PDEδ [32]. (C) Bound nucleotide in the KRAS structures. Majority of structures contain GDP and GNP is the most frequent from the non-hydrolysable GTP-analogues. The “Other” group include structures without nucleotides or appear with GDP/GTP competitive ligands. (D) Mutations in KRAS structures (HVR-only structures excluded). Almost half of the structures (46.5%) contain engineered mutations that are biologically irrelevant.

For long, the publicly available structural data of RAS proteins were dominated by HRAS. In 2011, most of the available RAS structures were HRAS (91%), as there were in total 99 HRAS, nine KRAS and one NRAS structures available in the Protein Data Bank (PDB) [31]. Only recently, as differences among RAS isoforms have been realised and the isoforms are not considered equal anymore, we have observed a resurgence in KRAS structures. As a result, on December 2019 there was 150 publicly available KRAS structures (Fig. 2A, Table1). From these, 144 structures are solved by X-ray crystallography and six structures are NMR-data driven models.

**Table 1:** PDB IDs of the publicly available KRAS structures. Entries are coloured based on the bound nucleotide (see Fig. 2C) and ordered by their mutations (see Fig. 2D) [4], [14], [21], [25], [32], [33], [34], [35], [36], [37], [38], [39], [40], [41], [42], [43], [44], [45], [46], [47], [48], [49], [50], [51], [52], [53], [54], [55], [56], [57], [58], [59], [60], [61], [62], [63], [64], [65], [66], [67], [68], [69], [70], [71], [72].

From all of the available KRAS structures, eight are HVR peptides co-crystallized with farnesyltransferases (Fig. 2B). In the remaining 142 structures, most are indeed lacking the flexible HVR region and represent only the G domain. The median size of these structures is 168 residues. More than half of the KRAS structures are GDP-bound and above third of all structures are bound to GTP or its non-hydrolysable analogue: GCP; GNP or GSP (Fig. 2C). Structures that are missing the nucleotide include HVR peptide structures, contain GDP/GTP competitive ligands or are from nucleotide exchange complexes (KRAS–SOS1).

Various mutations are present in the available structures. From the common oncogenic KRAS mutations, G12A, G12C, G12D, G12R, G12V, G13D and Q61H structures are represented (Fig. 2D). Additional engineered mutations are also quite common. Especially, the engineered C118S mutation is highly frequent (40%). C118 is the only cysteine residue in KRAS that is located on the protein surface. Moreover, in majority of the G12C structures, which contain a covalently bound ligand to C12, all other cysteine residues have been also mutated (C51S, C80L, C118S) (Fig. 2D, Table 1). Only in three structures with a C12 bound ligand, KRAS appears without additional mutations (PDB IDs: 5v71 [64], 5v9l [65], 5v9o [65]).

**Fig. 3:**
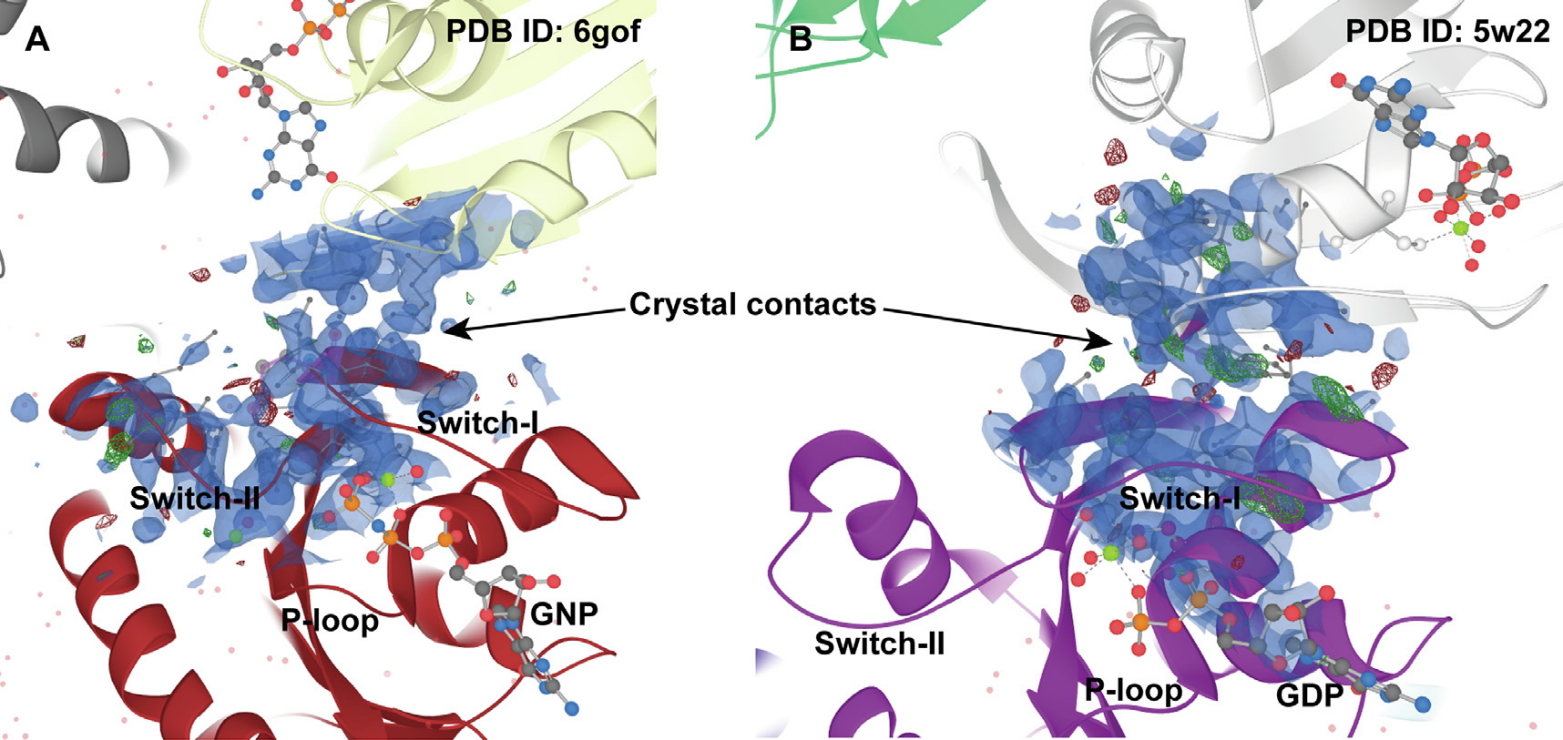
Crystal contacts stabilize the ordered switch regions. As an example, GNP bound G12D mutant (A) and GDP bound WT KRAS with engineered mutation C118S (B) are shown. Crystal contacts on top of the switch regions appear in various configurations among KRAS structures with ordered switches. Individual KRAS proteins depicted with cartoon in different colours. Electron density map, 2Fo-Fc σ = 1, is displayed in the switch region interface (blue). Images created with LiteMol [75] in PDBe [76]. (For interpretation of the references to colour in this figure legend, the reader is referred to the web version of this article.)

KRAS structural data may easily lead one to the false assumption that the switches appear in stabilized conformations. Only in quarter of available KRAS structures switch-I is disordered and in 37% switch-II is disordered (in one or more of the chains). In 19% of the structures both switch regions exhibit disorder. However, with a closer inspection of the crystal structures, it is quite evident that the switches, if not disordered, are stabilized via crystal contacts (Fig. 3). Various configurations of different crystal contacts are observed in structures that display ordered switch regions. To note, as the switch regions are in close proximity of each other and are connected via the beta-sheet β2–β3, the stabilization of one via crystal contacts may also affect the other. A crystal structure is always a spatiotemporal average of the protein structure, where crystal-packing contacts, which are crystal artefacts, may affect loop region conformations [73], [74]. In addition, stabilization of the switches may arise from crystallization conditions, as data acquisition for the X-ray crystallography-based model occurs typically in low temperature, further decreasing movement and dynamics of the protein. To point out, KRAS interacts with its effector proteins on top of these switch regions [5]; therefore, it is perhaps not surprising that KRAS tends to form protein–protein contacts in this interface also in the crystal environment. Nevertheless, distinct switch conformations are represented by the available structures. Only recently, structures displaying totally open switch-I conformation were published (PDB IDs: 6mqg [55], 6m9w [21], 6bof [68]). Generally, dynamics in switch-I and switch-II regions is not fully captured by the structural data.

## 3 Insights into KRAS dynamics from experimental data

Insights into the switch regions’ dynamical behaviour were first obtained via NMR spectroscopy [77]. Based on the results of NMR studies, it has been defined that switch-I appears in two distinct conformations, named as state 1 and state 2, where the former reflects to an open conformation and the latter to an closed conformation that is also found when RAS is in complex with an effector protein [78]. Initially these results were obtained by using HRAS proteins, but these states also occur in KRAS [46].

Interestingly, the GTP analogues, GCP and GNP (where the oxygen between the beta and gamma phosphate is a CH_2_ or NH, respectively), shift this switch-I conformational equilibrium towards state 1 [79]. These analogues are used in the majority of GTP-state mimicking crystal structures (Fig. 2C, Table 1). Moreover, the full length RAS shifts the equilibrium towards state 2, when compared to structures consisting only of the G domain [79].

Specific mutations in the switches, D33E in switch-I or A59G in switch-II, induce KRAS to crystallize in state 1 conformation, where switch-I is in an open conformation [58]. Also, NMR results indicated that replacing Y32 in switch-I to other amino acids shifts the equilibrium towards state 1 [79]. Of note, all RAS isoforms are identical in their amino acid composition in these regions. Thus, it seems that switch dynamics are quite sensitive to changes in their amino acid composition.

Although it is commonly stated in the literature that state-I is the *inactive* GTP-bound conformation, these D33E or A59G mutants display similar RAF-RBD (RAS binding domain) affinity as WT KRAS [58]. This perhaps highlights the fact that even though state 1 is not the end-point conformation of KRAS when bound to an effector protein, it may play a role in the association process of these protein–protein interactions. Therefore, state 1 should not be defined explicitly as an *inactive* KRAS state.

Recently, an additional layer of complexity to switch-region dynamics was identified, which provides another potential supplementary regulation mechanism of KRAS activity. The tyrosine residues Y32 and Y64, in switch-I and switch-II, respectively, can be phosphorylated via c-Src [80]. This phosphorylated state induces conformational changes in the switch regions and most likely traps KRAS into an inactive GTP-bound state, where a decreased affinity towards effector protein Raf-1 was observed. This switch-phosphorylation is reversible by SHP2 phosphatase, which is capable to dephosphorylate these tyrosine residues.

Not only are KRAS switch regions dynamic, but also a higher level rotational and translational dynamics exist in its native environment on the membrane, where the active KRAS signalling occurs [81]. The NMR-data driven models of KRAS on lipid nanodiscs revealed rotational complexity in KRAS’ membrane orientation [33]. These results suggested that KRAS occurs in occluded and exposed configurations on the membrane. These configurations were named based on the orientation of the effector protein binding interface of KRAS. In occluded configurations this interface is facing toward lipids and in exposed configurations it is pointing away from the membrane, allowing effector protein binding. To note, tethering of KRAS to the lipid nanodisc was achieved by maleimide-functionalized lipid (PE-MCC) at the C185 in its C-terminus and KRAS contained a C118S mutation. Regarding to translational dynamics of KRAS on the membrane, one of the main questions is the oligomerization state of KRAS. This is still somewhat unclear, as KRAS have been suggested to occur on the membrane as: monomer only [82]; monomers, dimers, oligomers [83]; dimer [84] or a trimer [85]. Overall, the data is still too scarce to understand the KRAS lateral mobility and the diffusion related to KRAS signalling [86].

## 4 Lessons from MD simulations

As experimental methods are still unable to fully describe protein dynamics, a deeper insight may be achieved by MD simulations [87]. Initial RAS MD simulations were conducted already in the 1990s. Although these simulations provided first insights to the RAS dynamics, they were too short to provide any reasonable insights into biologically relevant timescales in protein dynamics, which occur in microsecond timescale [88]. Additional issue with these simulations was the lack of available high-quality crystal structures at the time (Fig. 2A), for which especially the shorter simulations are more sensitive (as the starting configuration is decisive for the observations from short simulations). For this reason, these earlier RAS related simulations are not discussed here and the reader is recommended to read the comprehensive review by Prakash and Gorfe [89]. Simulations related to the enzymatic activity of RAS (GTP hydrolysis), mainly studied by QM/MM simulations, and simulations carried out with other methods than classical all-atom MD are out of the scope of this minireview (*e.g.* coarse grained or simulations conducted with enhanced sampling methods).

Kapoor and Travesset investigated different RAS isoforms’ (HRAS, NRAS and KRAS) dynamics with both of native ligands, GDP and GTP [90]. They simulated each individual system for hundred nanoseconds in two temperatures (300 K and 360 K) with four replicas, resulting in an aggregate of 5.46 μs simulation data. These simulations displayed high flexibility of the switch regions and that this flexibility was dissimilar among RAS isoforms. Since, it has been confirmed that the different isoforms exhibit distinct biochemical profiles [91]. These RAS simulations, however, were conducted with the G domain only, excluding the intrinsically disordered HVR, which indeed plays an important role in oncogenic signalling [92].

Simulations with the full-length KRAS with HVR in solution disclosed that the HVR may fold on top of the switch regions. This was first observed in simulations with the length of 100 ns [93], and later demonstrated in another study with 200 ns simulations in solution and also at the membrane [94]. These results are in agreement with the experimental NMR studies, as the observed shift with the full-length RAS in the equilibrium towards state 2 can be explained by the HVR folding on top of the switches in solution [79]. The oncogenic mutation influence to the full-length KRAS dynamics in solution was further investigated in total of 6.4 μs simulations, with the individual simulations lengths being 200 ns with two starting configurations (models) for each system [95]. Interestingly, some common oncogenic mutations, such as G12C, G12V and Q61H, displayed weakened HVR–G domain association. Furthermore, as there is a notable difference among the KRAS isoforms (see Introduction), an investigation of full length KRAS4A and catalytic domain only in solution with individual simulations of 100 ns length (total of 1.4 μs simulation time) suggested that KRAS4A is overall more dynamic when compared to KRAS4B [96].

KRAS’ rotational dynamics on the membrane have been also observed in the simulations where the membrane has been included. Prakash *et al.* demonstrated that KRAS appears in multiple distinct rotational conformations at the membrane with total simulation time around 8 μs, where individual replicas were simulated for 100–800 ns [97]. Comparably, KRAS4A simulations in diverse membrane environments with the length of 200–400 ns for individual systems (total of 5.8 μs) displayed distinct orientations at the membrane [98]. Recently, substantially longer microsecond timescales in the membrane simulations of KRAS were achieved. In a single 20 μs simulation of G12V mutant at the membrane three distinct conformations were observed [99]. Furthermore, G12D and Q61H mutants also displayed similar conformations in single 20 μs simulations, but also subtle differences how they populate these configurations [100]. Overall, based on these simulations it is quite clear that the monomeric KRAS displays rotational dynamic behaviour on the membrane, agreeing with the experimental data.

**Fig. 4:**
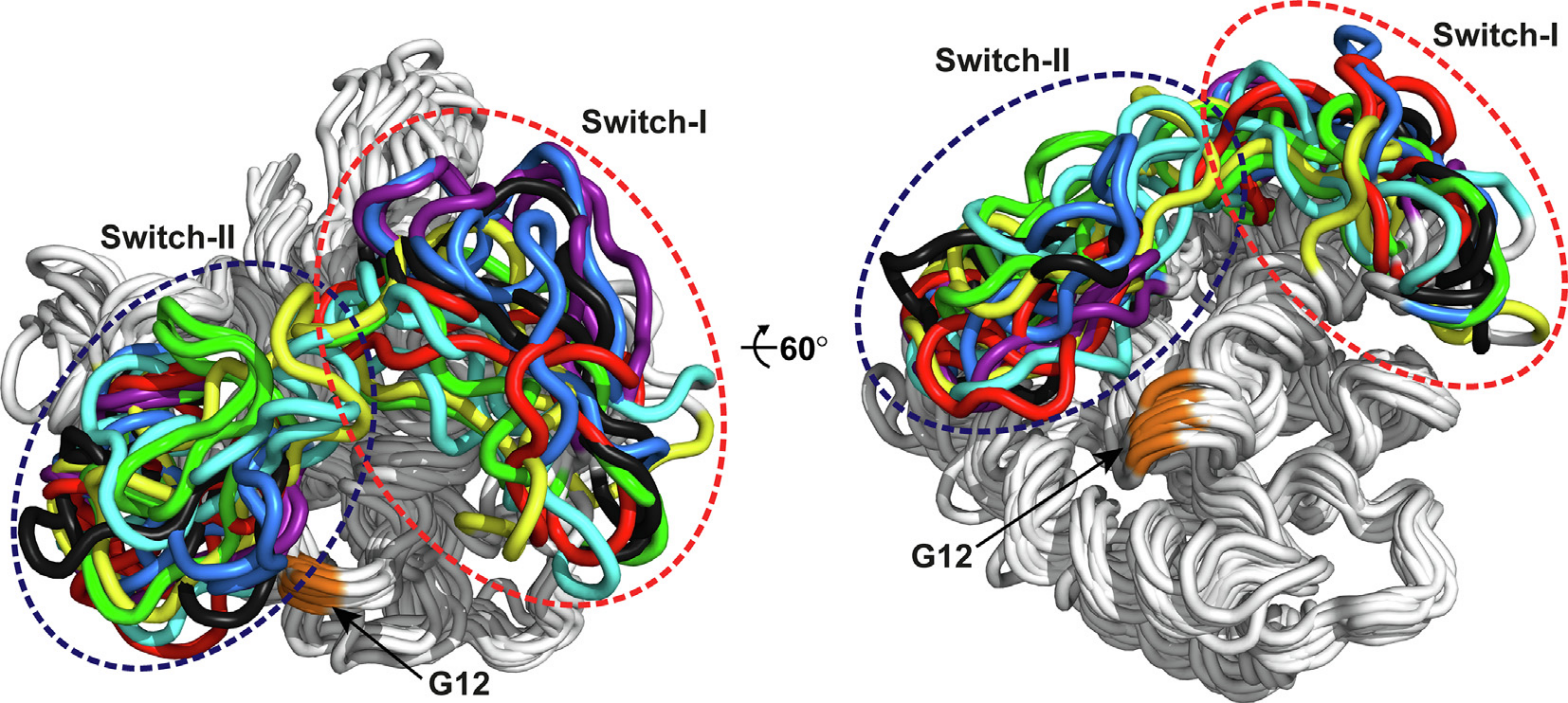
Metastable states of KRAS. The observed seven metastable states are mainly defined by different conformational ensembles of the flexible switch regions. Three conformations for each state are shown and the switches are coloured by individual colours for each state. Different G12 missense mutants populate these metastable states differently. For further details see Ref. [27]. (For interpretation of the references to colour in this figure legend, the reader is referred to the web version of this article.)

Differences in G domain dynamics of KRAS oncogenic mutants in solution have also been investigated. Sayyed-Ahmad *et al.* conducted single 1 μs simulations for WT KRAS, G12D, G12V and G13D mutants (and also for HRAS) [101]. Discrepancy among mutants in their dynamics was observed, which was highlighted by differences in residue contact probability networks. Also, local conformational shift of the G12D mutant compared to WT KRAS was observed in microsecond simulations (total simulation time of 8 μs) [102]. To assess differences among WT and selected oncogenic mutants (G12C, G12D, G12V, G13D and Q61H), Lu *et al.* conducted a total of 6.4 μs simulations with KRAS4B catalytic domain [103]. The individual systems’ trajectory lengths were between 200 and 400 ns. As the main result, an individual shift in the dynamics occurred by the mutants. To get a comprehensive picture of the putative position 12 missense mutant differences in their conformational dynamics, we simulated all KRAS G12 missense mutants with a total simulation time of 170 μs (85 individual 2 μs simulations) [27]. To note, this comprehensive sampling covered the cryptic state 1 conformations, excluding the closed state 2 conformation. Further analysis with Markov state models (MSMs), which allow assessment long-time statistical dynamics and transition probabilities of protein conformational ensembles (reviewed in [104]), revealed seven metastable states highlighting different conformational sub-states of the switches (Fig. 4). Notably, structural biology has been unable to capture all of these state configurations, and most likely will not be able to. These simulations also suggest that the state 1 should not be defined as a single conformation, which it is most often referred in the literature, but rather an ensemble of conformations. Strikingly, G12 missense mutants shift specifically KRAS dynamics, especially in the effector protein binding interface. For instance, the observed metastable states are populated differently, not only between WT and mutants, but also discrepancy among G12D, G12R and G12V mutants exist. Interestingly, G12D, which is the most frequent KRAS mutant in cancer, appears most similar in its dynamics compared to WT. These results indicate that the shift in KRAS dynamics occurs in allosteric manner and that a mutation can inflict changes in the protein dynamics in distant regions. Remarkably, we also observed highly opened short-lived switch-I conformations in the simulations, which should not be confused with metastable state conformations. Recent crystal structures of KRAS, which were published after these simulations, demonstrated extremely open switch-I conformations and thus support the validity of highly open switch configurations observed in simulations (PDB IDs: 6bof [68]; 6mqg [55]; 6mqn [55]; 6m9w [21]).

Not only have MD simulations brought insights to KRAS dynamics, but also our understanding of how KRAS binds and activates its effector proteins is starting to unravel. For instance, the KRAS mediated activation of PI3Kα occurs in allosteric manner [105], [106]. Here, the KRAS4B–PI3Kα-RBD interaction was investigated in solution with total of 10 μs simulations. Recently, KRAS interaction with Calmodulin was also investigated, with and without membrane in a total of 20 μs simulations [107]. These simulations suggest that the interaction between KRAS and Calmodulin is not static but rather dynamic, displaying various conformational ensembles.

## 5 Summary and outlook

The peculiar dynamics of the switches combined with intrinsically disordered HVR makes KRAS truly an enigmatic protein. Although the publicly available structural data of KRAS has been quickly evolving in the recent years, it is still far from complete. For instance, there are no structures available for the KRAS mutants G12S (7% of all KRAS G12X mutations observed in cancer), G13C (6% of all KRAS G13X mutations), Q61R and Q61K (19% and 8% of all KRAS Q61X mutations, respectively) (COSMIC v.90) [7]. Moreover, we are still lacking KRAS–effector protein functional complexes. Currently, we only have isolated pieces of the puzzle, which dramatically hinders our understanding of KRAS mediated signalling. To this end, a structure of more complete functional signalling complex, *i.e.* KRAS in complex with a full-length effector protein on the membrane, is required. This is most likely beyond X-ray crystallography and would require another method, *e.g.* Cryo-EM [108]. Furthermore, this type of structural information could provide insights to active KRAS’ oligomerization state, which would be extremely important, as there is emerging evidence that the nanoclustering and dimerization may play a significant role in oncogenic KRAS signalling [109].

Growing evidence is indicating that discrepancy among oncogenic KRAS mutants exist [4], [29], [30]. These putative subtle differences of the mutants and their differences in dynamics are experimentally difficult to address. For instance, even though the RAS mutants exhibit altered binding profile for the effector proteins, diminished or enhanced binding, a clear on/off binding changes are not observed [4], [110]. Overall, MD simulations have demonstrated that discrepancy among the mutants in their dynamics exist [27], [100], [103], especially in regions located in the effector protein binding interface, but these subtle differences and their biological consequences are still not properly understood.

Even though KRAS dynamics is extremely complex it should not be overlooked, as it could be the only way to gain proper understanding of its functions. This being said, more care should be put on the validity of the conducted MD simulations. Especially in the field of KRAS, where the underlying biology is so complex, overinterpreting MD simulation results is a true risk [111]. Furthermore, the sampling quality and the uncertainty quantifications of the future simulations should not be overlooked [112]. Even though breakthrough results are on high demand, simulations should never be conducted at the expense of the quality. To escape subjective observations from the trajectories, one should apply state-of-the-art methods, such as MSMs, to acquire more reliable long-time statistical dynamics of the biomolecules [113]. In addition, a great emphasis should be placed on careful planning to reach the timescale of conformational interest [88]. To achieve sufficient sampling, extra care should be taken in future as the system sizes increase due to inclusion of more components with the membrane to the simulations. For instance, diffusion events are slower with larger systems [114], [115], which implies that even longer simulation times are required with larger systems.

In future, the dynamics of KRAS dimeric and oligomeric complexes should be investigated in long timescale MD simulations. Moreover, the KRAS–effector protein association pathways would be important to study in more detail. Valid dynamics of these, however, might be difficult to capture before there is a clear support from the experiments ensuring that a structurally relevant complex is simulated.

To conclude, the understanding of KRAS structure and dynamics has a substantial role in deciphering its cryptic nature. The enhanced knowledge of these will aid us to connect the complex RAS biology to the molecular scale. These profound insights into KRAS functionality will support the drug discovery efforts against this difficult-to-drug target in future.

## Declaration of Competing Interest

The authors declare that they have no known competing financial interests or personal relationships that could have appeared to influence the work reported in this paper.
